# Exosomes in Cancer Radioresistance

**DOI:** 10.3389/fonc.2019.00869

**Published:** 2019-09-06

**Authors:** Jie Ni, Joseph Bucci, David Malouf, Matthew Knox, Peter Graham, Yong Li

**Affiliations:** ^1^Cancer Care Centre, St. George Hospital, Sydney, NSW, Australia; ^2^St. George and Sutherland Clinical School, Faculty of Medicine, University of New South Wales (UNSW) Sydney, Kensington, NSW, Australia; ^3^Department of Urology, St. George Hospital, Sydney, NSW, Australia; ^4^School of Basic Medical Sciences, Zhengzhou University, Zhengzhou, China

**Keywords:** cancer, exosomes, radiotherapy, bystander, radioresistance

## Abstract

Radiation is a mainstay of cancer therapy. Radioresistance is a significant challenge in the treatment of locally advanced, recurrent and metastatic cancers. The mechanisms of radioresistance are complicated and still not completely understood. Exosomes are 40–150 nm vesicles released by cancer cells that contain pathogenic components, such as proteins, mRNAs, DNA fragments, non-coding RNAs, and lipids. Exosomes play a critical role in cancer progression, including cell-cell communication, tumor-stromal interactions, activation of signaling pathways, and immunomodulation. Emerging data indicate that radiation-derived exosomes increase tumor burden, decrease survival, cause radiation-induced bystander effects and promote radioresistance. In addition, radiation can change the contents of exosomes, which allows exosomes to be used as a prognostic and predictive biomarker to monitor radiation response. Therefore, understanding the roles and mechanisms of exosomes in radiation response may shed light on how exosomes play a role in radioresistance and open a new way in radiotherapy and translational medicine. In this review, we discuss recent advances in radiation-induced exosome changes in components, focus on the roles of exosome in radiation-induced bystander effect in cancer and emphasize the importance of exosomes in cancer progression and radioresistance for developing novel therapy.

## Introduction

Cancer is a major health burden. Radiotherapy (RT) is widely used in more than 50% of localized cancer patients (1), and is a critical and inseparable component of comprehensive cancer treatment and care (2). In addition, RT is often combined with surgery, chemotherapy, and more recently, immunotherapy (3). Despite progress made in radiation delivery approaches and precision medicine, tumor therapeutic resistance and recurrence frequently occur in clinical settings.

Radioresistance is a complicated biological process associated with abnormal DNA damage response (DDR), apoptosis, autophagy, gene mutations, cell cycle checkpoint, and deregulated signaling pathways (4). It leads to poor prognosis in cancer patients and represents a major clinical obstacle for RT, which ultimately leads to tumor relapse and metastasis (5). Tumor microenvironment is an important factor affecting tumor progression and therapeutic response (6, 7). It was reported radioresistance is highly associated with tumor microenvironment (8, 9).

Exosomes are important components and regulators of the tumor microenvironment. Exosomes are small extracellular vesicles (40–150 nm) secreted by different cells. Exosomes are representative of the original cells and can reflect a regulated sorting mechanism (10). These vesicles are composed of proteins (receptors, transcription factors, enzymes), nucleic acids [nuclear DNA, mitochondrial DNA (mtDNA), mRNA, microRNA (miRNA), long non-coding RNA (lncRNA), and circular RNA (circRNA)], and lipids (11–14). Exosome cargos such as nucleic acid and proteins from originating tumor cells communicate with neighbor cells or recipient cells, resulting in cancer progression and recurrence (15, 16).

Stressful conditions affect exosome secretion, composition, abundance, and potential binding on recipient cells. It was reported that different physiological and environmental conditions could alter the composition of exosomes shed from cells (17). Accumulating evidence indicates an increased release of exosomes after exposure to RT and the altered contents from donor cells were more oncogenic (18–21). Several recent studies have confirmed irradiated cells were involved in radiation-related communication between cells (20, 22, 23).

Despite the progress that has been made in exosome-mediated functions in *in vitro* models, there are still many challenges to be faced in exosomes and radiation oncology research. Here, we review recent advances in the radiation-induced exosome changes, discuss the roles of exosome in radiation-induced bystander effect (RIBE) in cancer and emphasize the importance of exosomes in cancer radioresistance and progression for the development of novel therapeutic strategies.

## Radiation-Induced Exosome Changes in Cancer Radiotherapy

Cellular stress affects the composition and abundance of exosomes, as well as their potential impact on the recipient cells. Exosomes are a major environmental factor for cellular stress, and radiation can enhance the release of exosomes and affect exosome-based intercellular communication, which has been observed in various types of normal and tumor cell lines (18, 19, 24).

An increased level of exosomal CD276 was observed in the irradiated and senescent 22RV1 prostate cancer cell line, suggesting that this marker may provide a non-invasive way to monitor the efficacy of RT for prostate cancer patients (25). It was reported that circulating Hsp72 level was increased after radiation exposure in prostate cancer mouse xenografts and clinical sample; and that the exosomes containing Hsp72 are a possible contributor, leading to pro-inflammatory cytokine production and immune modulation (26). Khan et al. found that the level of exosomal survivin was increased after proton irradiation in HeLa cells and the rate of exosome secretion was not influenced (27). These findings suggest exosomal survivin may be associated with cancer recurrence after RT and could be a potential therapeutic target for preventing cervical cancer progression.

Radiation also alters the molecular composition within the exosomes. In one study, it was found radiation increased levels of exosomal connective tissue growth factor (CTGF) and Insulin Like Growth Factor Binding Protein 2 (IGFBP2) proteins, both of which are important in cell migration. By transferring CTGF mRNA, exosomes from irradiated cells were found to upregulate the migration-related signaling molecules including neurotrophic tyrosine kinase receptor type 1, focal adhesion kinase, Paxillin, and proto-oncogene tyrosine-protein kinase Src in recipient cells (18). In another study, exosomes released from the irradiated head and neck squamous carcinoma cell (HNSCC) FaDu cells demonstrated a distinctive protein expression profile compared to those from non-irradiated cells (20). Interestingly, most of the proteins that are specifically overexpressed in exosomes are those involved in transcription, translocation, and cell division, indicating that exosomal cargo is reflective of radiation-induced changes in cells (20). Similarly, exosomes derived from irradiated HNSCC BHY cells were found to be associated with not only immunity but also cell adhesion and motility, the underlying molecular mechanism being enhanced AKT-signaling triggered by the exosomal proteins (28). Using a shotgun liquid chromatography–tandem mass spectrometry (LC-MS/MS) approach, Abramowicz et al. found 472 exosomal proteins that are significantly affected by ionizing radiation (IR) in HNSCC UM-SCC6 cells and identified their role in mediating the cellular response to IR (29). Zhao et al. recently identified 63 upregulated and 48 downregulated circular RNAs from the exosomes of radioresistant glioma cells compared with those in control cells. Using qRT-PCR, they found circATP8B4 from radioresistant exosomes of glioma cells may be transferred to radiation-naïve cells and promoted cell radioresistance by acting as a microRNA(miR)-766 sponge (30).

The role of exosomes in radiation has garnered increasing attention in recent years. One study screened 752 exosome-derived miRNAs of locally advanced non-small cell lung cancer (NSCLC) patients and demonstrated that increased radiation dosage reduced miR29a-3p and miR150-5p expression (31), indicating that circulating exosomal miRNAs could help predict RT toxicity. In another study evaluating the outcome of HNSCC patients treated with chemoradiation therapy (CRT), exosomes from pooled plasma samples of patients who had complete or incomplete responses to CRT were screened. They identified a distinctive expression pattern of proteins between patients who had a complete response and who did not (32). In a recent Phase I clinical trial, 18 HNSCC patients receiving a combination of cetuximab, ipilimumab and RT were serially monitored for tumor cell-derived exosomes and T cell-derived exosomes. The results suggested tumor cell-derived exosomes and T cell-derived circulating exosomes instead of immune cells were suitable for monitoring of patients' responses to oncological therapy, supporting the potential role of exosomes as a non-invasive tumor and immune cell biomarkers in cancer (33).

The exosomes in RT research is stating to move from bench to bedside. However, the main limitation is the sample numbers tested from patiensts are still low and the preliminary results obtained need to be further validated in a large cohort of patients. Another challenge is the methods used for detecting exosome contents (biomarkers) such as proteomics and next generation sequencing need to be further optimimised to obtain the maximum number of interesting pontential biomarkers for verification. Although advances in exosome-based biomarkers such as proteins and miRNAs highlight an optimistic outlook for RT, the great progress has not been achieved due to limited reports in clinical research. Future direction in this area should move on the translational research and clinical trials.

In summary, the studies so far on radiation-induced changes in exosome composition have mainly been confined to their proteome contents using *in vitro* cancer cell line models (Summarized in Table 1), and there is a big gap in understanding how these changes are regulated, with the hope of translating into their functional importance. Future studies in this area should focus on (1) investigating the mechanisms of how the components of exosomes from donor cells after radiation are transferred to receipt cells; (2) establishing *in vivo* animal models for further studying the changes of exosomal components on the effect of RT; (3) investigating whether the changes of exosomal components could be used as biomarkers to evaluate the efficacy of RT; and (4) investigating whether the changes of exosomal components could be used as useful therapeutic targets to overcome radioresistance and improve the current RT.

**Table 1:** Changes of exosomal components in cancel cell lines *in vitro* after radiation treatment.

**Source of exosomes**	**Study model**	**Dose of IR**	**Time from IR to exosome isolation**	**Changed components**	**Test approach**	**Isolation method**	**Effect**	**References**
22RV1 cell line	Human CaP *in vitro*	4 Gy	4 days	CD276	SDS-PAGE	UC	Exosomes can transfer cargos with both immunoregulatory potential and genetic information	(25)
HeLa cell line	Human cervical carcinoma *in vitro*	3 Gy	24 h	Survivin	Western blotting, LC-MS/MS	UC	Exosomes represent a novel secretory pathway by which proteins can be actively released from cells.	(27)
LN18, U87MG, U251cell lines	GBM *in vitro*	2 to 8 Gy	12 to 48 h	CTGF mRNA and IGFBP2	Immunoblot analysis, qRT-PCR	UC	Radiation influences exosome abundance, specifically alters their molecular composition.	(18)
FaDu cell line	HNSCC *in vitro*	2Gy	18 h	Proteins (*n* = 236)	LC-MS/MS	Total exosome isolation kit	Exosomal cargo reflected radiation-induced changes in cellular processes like transient suppression of transcription and translation or stress-induced signaling.	(20)
BHY cell line	HNSCC *in vitro*	6 Gy	24 h	Up-regulayed (*n* = 39) and downregulated *n* = 36) proteins	LC-MS/MS	UC	Exosomes may act as driver of HNSCC progression during radiotherapy.	(28)
UM-SCC6 cell line	Human HNSCC *in vitro*	A single 2, 4 or 8 Gy	24 h	Proteins (*n* = 472)	LC-MS/MS	SEC	The protein profiles of exosomes released by radiaton were established.	(29)
RR-U251cell line	Human GBM cell line *in vitro*	60 Gy	N/A	circATP8B4	RNA sequencing	UC	circRNA acts as a miRNA sponge, which may regulate tumor radioresistance.	(30)

*CaP, prostate cancer; CTGF, connective tissue growth factor; HNSCC, head and neck squamous cell carcinoma; GBM, glioblastoma; IGFBP2, insulin-like growth factor binding protein 2, LC-MS/MS, liquid chromatography-tandem mass spectrometry; N/A, not applicable; SEC, size-exclusion chromatography; UC, ultracentrifugation*.

## The Bystander Effects by Radiation-Induced Exosomes

Radiation affects not only its direct targeted cells but also non-irradiated neighbors. This is evidenced by RIBE that cells that were not exposed to radiation exhibit effects as a result of intercellular communication. RIBE can also lead to biological changes in bystander cells and tissues, including chromosomal rearrangement, genomic instability, DNA damage, gene expression alteration, and apoptosis (34).

Numerous studies have demonstrated a large and complex interconnected web of mechanisms that contribute to the generation of RIBE, including reactive oxygen species (ROS), cytokines, free radicals, immune system, and epigenetic modulators (35–37). As previously reviewed more than a decade ago, it was believed that RIBE was mediated by both “a soluble secreted factor” and the cell-to-cell gap junction (38). It was not until recently that the important role of exosomes mediating RIBE had been recognized (39). Exosomes shed by irradiated cells are putatively involved in different aspects of the systemic response to IR, including the RIBE (40–42). Jella et al. showed that exosomes released from gamma-irradiated keratinocyte HaCaT cells induced increased cell death and ROS production in non-irradiated cells (24).

Mechanistically, IR elicits a set of dysregulated proteins and nucleic acids within the cell. These effectors, such as proteins, miRNAs and mtDNAs, are packaged into exosomes during their formation, which are then released to the extracellular environment. These cargos within the radiation-targeted cell-released exosomes subsequently get access to the adjacent cells as a result of exosome migration and internalization, and prompt RIBE in the non-targeted distant cells (21, 43–45).

These components of exosomes have different functions in RIBE, such as regulation of inflammation and modulation of DDR (46).

One report demonstrated that the production and release of exosomes following radiation-induced DNA damage were regulated by the p53 pathway (47). Tian et al. demonstrated that miR-21, a well-established DDR-related miRNA, played a mediating role in bystander DNA damage since it elevated ROS levels and increased the double-strand break (DSB) marker p53-binding protein 1 (53BP1) foci in non-irradiated cells (48). The role of miR-21 in RIBE was also validated by Yin et al. and Xu et al. using different experiment models (21, 49). Exosomal miR-1246 was also found to act as a messenger and contribute to DNA damage by directly repressing the DNA Ligase 4 (LIG4) gene (22). In combination, Yentrapalli et al. found that proteins such as afamin and serpine peptidase F1 along with miRNAs including miR-204-5p, miR-92a-3p, and miR-31-5p, play an important role in inducing and regulating RIBE (50). Another group also demonstrated that exosomal proteins and RNAs could mediate short- and long-term RIBE in human MCF-7 breast cancer cells (19, 51), implying that proteins and miRNAs may work synergistically during this process.

**Figure 1:**
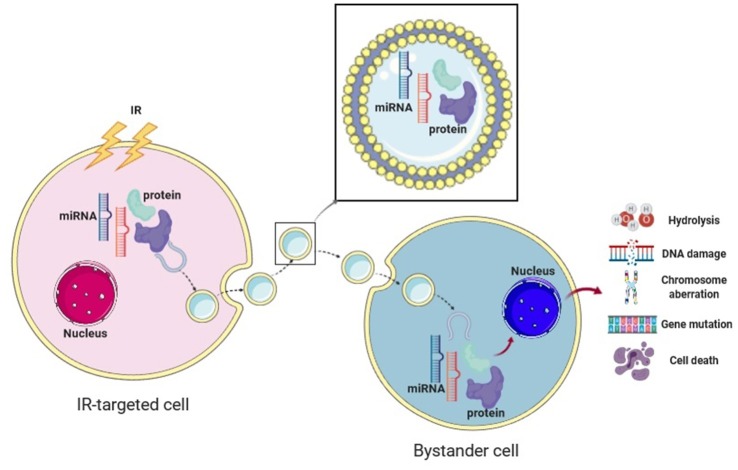
The bystander effect of exosomes in cancer radiotherapy. The mechanistic diagram displaying the effect of radiation-induced exosomal proteins and microRNAs in mediating cellular and molecular changes in RIBE. Created with BioRender.

Moreover, exosomal miRNAs are able to travel remotely to influence cellular functions and regulate the niche-host reaction in targeted or non-targeted cells. It is worth mentioning that the role of a miRNA as either a positive or negative regulator in the RIBE varies from cell type to cell type. Interestingly, cells affected by RIBE and their progeny also showed the ability to secrete exosomes, and this cascade could potentially lead to a delayed RIBE-related inflammatory response (19). We have summarized exosome-mediated RIBE studies in cancer RT in Table 2. The mechanistic diagram demonstrating effects of radiation-inducible exosomal miRNAs and proteins in mediating RIBE is shown in Figure 1.

**Table 2:** Summary of RIBEs caused by exosomes in recipient cells.

**Donor cells**	**Recipient cells**	**Dose of IR**	**Time from IR to exosome isolation**	**Isolation method**	**RIBEs in receipt cells**	**Effect**	**References**
Human non–small cell lung cancer cell lines (H460, H1299)	H460, H1299	5 Gy	48 h	UC	p53-dependent response to DNA damage (Western blot) after 24 h of incubation.	The p53 pathway regulates the production of exosomes for cancer communication	(47)
Human breast cancer cell line (MCF-7)	MCF-7	2 Gy	4 h	UC	DNA (comet assay) and chromosomal (metaphases analysis) damage after 24 h of incubation	Exosomes are partially involved in the bystander effect and genomic instability	(51)
Human breast cancer cell line (MCF-7)	MCF-7	2 Gy	4 h	UC	DNA (comet assay) and chromosomal (metaphases analysis damage) after 24 h of incubation up to 20 cell-doublings	Exosomes are associated with signaling of the non-targeted effects (NTE) of IR, initiated by both exosomal RNA and protein molecules.	(19)
Human HNSCC cell lines (BHY, FaDu)	BHY, FaDu	0–9 Gy	24–48 h	UC	Increase uptake of exosomes and survival and affect rates of DNA double-strand break repair after 24–48 h of incubation	Exosomes transmit prosurvival effects by promoting the proliferation and radioresistance	(52)
Human papillomavirus–immortalized human bronchial epithelial (BEP2D),	BEP2D	2 Gy	4–8 h	UC	miR-1246 packaged in exosomes affecting DNA damage by directly repressing the LIG4 gene after 24 h of incubation	Exosomal microRNAs have potentials as a messenger and contribute to DNA damage by directly targeting mRNA.	(22)

*h, hours; HNSCC, head and neck squamous cell carcinoma; UC, ultracentrifugation*.

In the therapeutic arena, on the one hand, RIBE is harmful to normal tissues, but on the other hand may be beneficial to induce non-irradiated cancer cell death during the treatment. RIBEs have critical implications in cancer RT. As direct effects of RT and RIBE are mechanistically distinctive and therefore it is important to develop different types of drugs to specifically target each mechanism, such as novel radiosensitisers to upregulate RIBE to kill more adjacent tumor cells; or adjuvant inhibitors to minimize the RIBE-induced systemic toxicity after RT.

Moreover, although *in vitro* studies bring promise for using exosomes and their cargo to regulate DDR and RIBE, it remains unclear whether these findings can be translated into the clinical setting. In future studies, the mechanisms of RIBE need to be deeply investigated, and *in vivo* animal models and clinical samples should be applied.

## Exosomes in Cancer Progression After Radiotherapy

RT itself may increase the motility of surviving cancer cells, evidenced in glioblastoma (GBM), lung cancer and HNSCC, thus facilitating the spread of the tumor to local and distant sites (53–55). Radiation-induced exosomes have recently found to be an accomplice in promoting tumor cell motility and assisting in the pre-metastatic niche formation, the effectors again being the exosomal cargo incorporated by the recipient cells. Arscott et al. showed that radiation-derived exosomes enhanced U87MG GBM cell migration in co-culture (18). Using a wound healing assay, Mutschelknaus et al. found a pro-migratory role of exosomes in boosting the migratory capacity of BHY and FaDu HNSCC cells, in a dose-dependent and AKT-dependent manner (28). Apart from cell motility, angiogenesis also plays a crucial role in RT and tumor metastasis. Zheng et al. recently demonstrated that in lung cancer, the exosome-induced pro-angiogenesis effect was enhanced when the A549 and H1299 lung cancer cells were exposed to IR, and the miR23-mediated phosphatase and tensin homolog (PTEN) downregulation played an important role in this process (56). These findings indicate that radiation-induced exosomes function as a driver of cancer progression and metastasis during RT, and may represent a putative target to improve RT strategies.

The recent striking responses to immune-checkpoint inhibitors in the treatment of melanoma and solid tumors are paradigm-shifting and stirring up much research interest in the combination of immune-checkpoint inhibitors with RT. There is a close association between radiation response and immunity (57). RT plays either an immune-suppressive role (due to the sensitivity of leukocytes) or an immune-stimulatory role, evidenced by enhancing several antigen processing and presentation pathways (58, 59). Conventionally, the immune stimulation is believed to be T cell-mediated, but it is not until recently have we found that T cell-derived exosomes also plays a role in promoting esophageal cancer metastasis, via activation of epithelial-mesenchymal transition (EMT), β-catenin, and NF-κB/SNAIL pathways (60). This study provides a rationale for targeting exosomes during the synergy between RT and immunotherapy, but there are still quite a few unsolved riddles in this synergy, such as the sequencing, dose, and fractionation. Clearer mechanistic understandings of RT's immune-stimulatory role and further investigations on exosomes' functions in immune modulation are needed to expand this research field.

## Exosomes in Radioresistance

Despite the recent advances in RT, many cancer patients, especially the locally advanced ones, failed radiation treatment (radioresistance), leading to a local recurrence or even distant metastasis. As previously reviewed (4), radioresistance can arise either from genetic or phenotypic changes within the tumor or as a result of the tumor stromal and microenvironment protecting the tumor against IR. Exosomes are one of the key components of the tumor microenvironment and increasing evidence suggests that they play a significant role in facilitating the development of radioresistance.

The key players in exosome-mediated radiosensitivity are found to be exosomal non-coding RNAs, proteins, and the crosstalk with survival and apoptotic pathways. Mutschelknaus et al. recently demonstrated that exosomes derived from irradiated HNSCC cells transmitted pro-survival signals to recipient cells via exosome cargos (52). In breast cancer, RNAs within exosomes were found to regulate radioresistance via an antiviral STAT1/NOTCH3 pathway (61). Radiation-induced exosomal miR-208a increased the proliferation and radioresistance via targeting p21 with activation of the AKT/mTOR pathway in lung cancer (62). In GBM, Mrowczynski et al. recently discovered that exosomes could enhance cell survival to radiation exposure by increasing levels of oncogenic miRNAs, mRNAs and pro-survival pathway proteins and at the same time decreasing levels of tumor-suppressive miRNAs and mRNAs (23). Recently, other exosomal non-coding RNAs were also found to be involved in the promotion of radioresistance, such as long non-coding RNA AHIF in glioblastoma (63), and circATP8B4 in glioma (30).

While exosomes were reported to play an important role in the promotion of cancer radioresistance, other reports were controversial. Wang et al. recently reported that autocrine secretions enhance the radioresistance of H460 NSCLC cell line in an exosome-independent manner and that these secretions mainly affect the DNA repair process (64). In another study, it was shown that exosomes derived form mesenchymal stem cells (MSCs), combined with RT, enhance RT-induced cell death in tumor and metastatic tumor foci in a melanoma mouse model. The finding provides a rationale to use MSCs-derived exosomes as an adjuvant to support and complement RT (65). These data indicate the role of exosomes in cancer radioresistance is complicated and could be affected by many factors such as tumor type, tumor microenvironment, experimental methods or different combinations of therapies.

Cancer is highly heterogeneous and includes a small subset of cells that possess the capacity of self-renewal and differentiation, referred to as cancer stem cells (CSCs) (66). CSCs are inherently more resistant to radiation than ordinary cancer cells, and more likely to survive after being exposed to RT. On one hand, these surviving CSCs may release exosomes to transfer resistant or refractory phenotypes to recipient cells, limiting the treatment efficacy. It was found that lncRNA H19 in exosomes derived from CSCs induced angiogenesis in hepatocellular carcinoma (67). Exosomes released from prostate and breast CSCs were also capable to induce autophagy (68), which has been shown to modulate sensitivity of cancer to RT (69). On the other, CSCs can also be eliminated via being reprogrammed into non-tumorigenic cells, using exosomes derived from adipose-derived stem cells (70). It not only re-justifies that the CSCs have to be eradicated during cancer therapy, but also preludes the usage of exosomes with modified surface or cargos to target CSCs.

**Figure 2:**
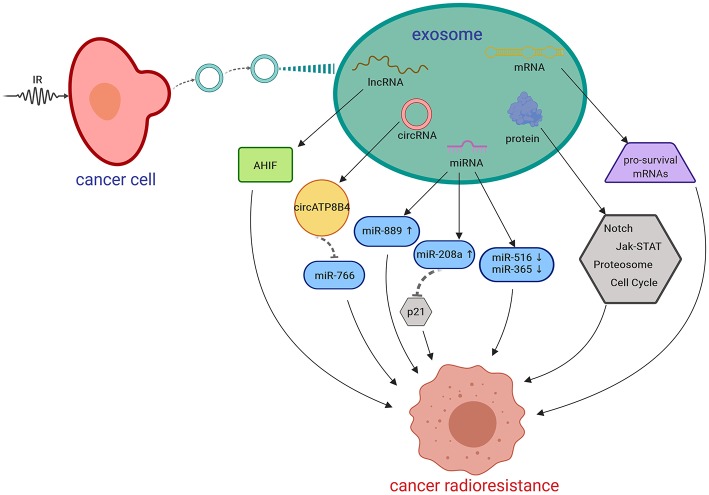
Putative mechanisms of IR-induced exosomes in promoting cancer radioresistance. AHIF, a natural antisense transcript; circRNA, circular RNA; IR, irradiation; lncRNA, long non-coding RNA; miRNA, microRNA; mRNA, messenger RNA. Created with BioRender.

In summary, all the data indicate that radiation-derived exosomes play important roles in cancer radioresistance through re-programmed cargos and intercellular communication. mRNAs, non-coding RNAs and signaling pathway proteins are closely related in exosome-associated radioresistance. CSC-associated exosomes are also a potential player in radioresistance and should be deeply investigated in the future study. The potential mechanisms of exosomes in radioresistance are shown in Figure 2.

## Conclusions

Exploiting the biological functions of exosomes is intriguing, as they provide a snapshot of the entire tumor, transfer molecules intercellularly and can be used as a therapeutic target. In order to achieve these goals, isolation of exosomes should be standardized and optimized.

Exosomes in radiation research is a new and developing area. Radiation affects not only the production of exosomes but also the composition within, which makes exosomes an ideal prognostic and/or predictive biomarker to monitor the radiation response.

The radiation-altered exosomal cargos can be taken up by recipient cells, thus exerting various biological functions to impact radiosensitivity, and it is also worthwhile to note that this impact can be the results of synergistic or opposing effects of different exosomes for the sake of their heterogeneity. Due to the unique physical and biological features of exosomes, using functional exosomes to target CSCs and facilitate immunotherapy, is a promising avenue to be explored in RT, since exosomes are more stable, endogenous, and can be easily engineered or labeled.

There are still many challenges existing for exosome study. The future development of methodologies for exosome isolation and purification should be universal, precise, and suitable for clinical settings. Our experience is the combination of two or more approaches is a better choice for exosome isolation. In addition, the optimal sample source should be determined based on the cancer types and the preservation conditions of samples should be standardized. Furthermore, specific biomarkers of tumor exosomes should be screened and exploited for investigating the mechanisms of radioresistance.

Currently, knowledge of exosomes in cancer RT is in its infancy and mostly limited in *in vitro* studies. Further *in vivo* and clinical studies are warranted. Increasing knowledge of the biology of exosome and its cargo, along with standardized methods for exosome isolation and characterization will greatly contribute to a better understanding of mechanisms of exosome-mediated RT response and a good harnessing of exosomes as a therapeutic target, which might ultimately lead to the development of novel treatment strategies.

## Author Contributions

JN, JB, DM, MK, PG, and YL reviewed the literature, developed the structures, wrote the review, and approved the final manuscript.

### Conflict of Interest Statement

The authors declare that the research was conducted in the absence of any commercial or financial relationships that could be construed as a potential conflict of interest.
